# Nutrition in Perinatal Midwifery Care: A Narrative Review of RCTs, Current Practices, and Future Directions

**DOI:** 10.3390/healthcare14020283

**Published:** 2026-01-22

**Authors:** Artemisia Kokkinari, Maria Dagla, Kleanthi Gourounti, Evangelia Antoniou, Nikoleta Tsinisizeli, Evangelos Tzamakos, Georgios Iatrakis

**Affiliations:** Department of Midwifery, School of Health & Care Sciences, University of West Attica, 12243 Athens, Greece; mariadagla@uniwa.gr (M.D.); kgourounti@uniwa.gr (K.G.); lilanton@uniwa.gr (E.A.); nikoletatsinisizeli@gmail.com (N.T.); tzamvagpal@uniwa.gr (E.T.); giatrakis@uniwa.gr (G.I.)

**Keywords:** perinatal nutrition, breastfeeding nutrition, midwifery care, pregnancy diet, perinatal health, pregnancy diet, lactation, maternal health, neonatal outcomes, nutritional counseling, public health

## Abstract

Background: Nutrition during the perinatal period, including pregnancy, childbirth, postpartum, and lactation, is a critical determinant of maternal and neonatal health. While the importance of balanced nutrition is well established, the integration of nutritional counseling into midwifery care remains inconsistent across settings. Evidence suggests that midwives are uniquely positioned to deliver nutrition-related support, yet gaps persist in their formal training and in the availability of structured guidance. These gaps are particularly evident in certain regions, such as Greece, where dedicated national guidelines for perinatal nutrition are lacking. Methods: This systematized narrative review synthesises evidence from studies published between 2010 and 2025, retrieved through PubMed, CINAHL, Scopus, and relevant national guidelines. Although the synthesis draws on diverse study designs to provide contextual depth, randomized controlled trials (RCTs) were prioritized and synthesized separately to evaluate the effectiveness of midwife-led interventions. In total, ten randomized controlled trials were included in the evidence synthesis, alongside additional observational and qualitative studies that informed the narrative analysis. Both international and Greek literature were examined to capture current practices, challenges, and knowledge gaps in the nutritional dimension of midwifery care. Results: Findings indicate that adequate intake of macronutrients and micronutrients, including iron, folic acid, vitamin D, iodine, calcium, and omega-3 fatty acids, is essential for optimal maternal and neonatal outcomes. Despite this, studies consistently report insufficient nutritional knowledge among midwives, limited confidence in providing counseling, and variability in clinical practice. Socio-cultural factors, such as dietary traditions and migration-related challenges, further influence nutritional behaviors and access to guidance. Emerging approaches, including e-health tools, group counseling models, and continuity-of-care frameworks, show promise in enhancing midwives’ capacity to integrate nutrition into perinatal care. Conclusion: Nutrition is a cornerstone of perinatal health, and midwives are strategically placed to address it. However, gaps in training, inconsistent guidelines, and cultural barriers limit the effectiveness of current practices. Strengthening midwifery education in nutrition, developing context-specific tools, and fostering interdisciplinary collaboration are essential steps toward more comprehensive and culturally sensitive perinatal care. Future research should focus on longitudinal and intervention studies that assess the impact of midwife-led nutritional counseling on maternal and neonatal outcomes.

## 1. Introduction

Nutrition during the perinatal period plays a critical role in shaping maternal and neonatal health outcomes, influencing metabolic, immunological, and developmental trajectories across the life course. Nutrition during pregnancy, childbirth, and lactation is a key determinant of both maternal well-being and neonatal outcomes, influencing growth, immune development, and long-term health trajectories [1]. While clinical aspects of midwifery care traditionally focus on monitoring pregnancy and delivery, midwives are increasingly recognized as frontline providers of health education, prevention, and counseling, with a unique position to address nutritional issues across diverse populations [2].

Despite this importance, recent evidence emphasizes that key aspects of women’s health, particularly nutrition during pregnancy, childbirth, and the postpartum period—remain underrepresented in research, limiting the development of context-specific and gender-sensitive nutritional frameworks [3]. This knowledge gap is particularly critical in contexts such as Greece, where national frameworks emphasize general dietary advice but lack perinatal-specific guidelines for pregnant and lactating women [4]. Addressing these gaps is essential for ensuring consistent, evidence-based nutritional support throughout the perinatal period.

International evidence underscores that perinatal nutrition has long-term implications not only for immediate pregnancy outcomes but also for the future health of both mother and child. Deficiencies in key micronutrients such as folate, iron, iodine, and omega-3 fatty acids have been linked to increased risks of preterm birth, low birth weight, impaired neurodevelopment, and postpartum depression [1]. Recent evidence from systematic reviews and meta-analyses indicates that maternal dietary patterns such as the Mediterranean diet are associated with lower risks of gestational diabetes, preeclampsia, preterm birth, and adverse neonatal outcomes, emphasizing the impact of overall diet quality on long-term maternal and child health [5]. Additionally, the World Health Organization’s (WHO) 2023 [6] nutrition counselling recommendations highlight the importance of antenatal nutrition education and counselling to improve maternal nutritional status and perinatal outcomes across diverse settings. However, the extent to which these risks are mitigated through structured dietary counseling remains inconsistent across health systems.

Comparative studies reveal significant differences in midwives’ knowledge and confidence in providing nutrition-related advice. For example, a scoping review of perinatal care providers found that many midwives recognized the importance of nutrition counseling but reported insufficient training and limited access to updated resources, which reduced their ability to offer tailored guidance [2]. This aligns with findings from population-level reviews showing that underrepresented or socioeconomically disadvantaged groups are disproportionately affected by poor maternal nutrition, further highlighting the need for equitable and culturally sensitive care models [3].

Evidence from continuity-of-care models, where midwives provide consistent support across pregnancy, birth, and the postpartum period, indicates improved maternal satisfaction and, in some settings, enhanced neonatal outcomes compared to fragmented care systems [7,8]. These models may also provide an optimal framework for embedding structured nutrition counseling as part of routine care. Within the Greek healthcare system, however, the implementation of continuity-of-care models remains limited, largely due to the fragmented organization of maternal services and the historically underdeveloped role of primary care in coordinating perinatal care. Analyses of the Greek health system highlight that strengthening primary healthcare and integrating midwife-led services could provide an appropriate structural platform for continuity-based models, including sustained nutritional counselling across the perinatal continuum [9]. Yet, in many countries, including Greece, the integration of nutrition into midwifery practice is hampered by the absence of perinatal-specific dietary guidelines [4].

Midwives are uniquely positioned within antenatal care to support maternal nutrition by facilitating the translation of evidence-based recommendations into routine clinical practice [2,10].

Beyond meeting increased energy demands, maternal nutrition during pregnancy exerts a direct influence on pregnancy complications and neonatal outcomes. Suboptimal dietary quality has been strongly associated with higher risk of gestational diabetes mellitus (GDM). For example, Zhang et al. [11] reported that women with high intake of processed carbohydrates and sugar-sweetened beverages exhibited a significantly greater risk of GDM compared to those adhering to diets rich in whole grains and vegetables. Similarly, maternal diets dominated by saturated fats have been linked to increased rates of preeclampsia and excessive gestational weight gain, both of which are associated with adverse neonatal outcomes such as preterm birth and macrosomia [12,13,14].

Micronutrient deficiencies have equally important implications. Maternal iron deficiency not only increases the likelihood of anemia but has also been associated with higher rates of cesarean section and low Apgar scores in neonates [15]. Folate insufficiency, if uncorrected, leads to neural tube defects, while inadequate vitamin B12 may exacerbate risks of intrauterine growth restriction (IUGR) [16]. Deficient vitamin D status in pregnancy has been associated with impaired glucose tolerance [17], higher risk of preeclampsia [18], and impaired skeletal development in the fetus [19].

In contrast, balanced dietary patterns such as the Mediterranean diet have shown protective effects during pregnancy. Adherence to a Mediterranean dietary pattern, characterized by high intake of fruits, vegetables, whole grains, fish, and olive oil, has been associated with lower risks of GDM, hypertensive disorders, and preterm delivery [5,20,21]. Importantly, such dietary approaches are feasible in Mediterranean countries, though cultural and socioeconomic barriers may limit consistent adherence.

The role of the midwife in this context becomes even more critical. By integrating evidence-based nutritional counseling into antenatal care, midwives can help women shift from high-risk dietary habits toward protective patterns. Evidence from randomized controlled trials suggests that midwife-led nutritional interventions can significantly reduce gestational weight gain and improve maternal metabolic outcomes, with positive effects extending into the postpartum period [22].

Calcium is fundamental during pregnancy for fetal skeletal mineralization and maternal bone health. Inadequate intake has been linked to increased maternal bone resorption and higher risk of hypertensive disorders, particularly preeclampsia. Beyond nutritional factors, appetite may also be suppressed by the use of substances such as alcohol, cigarettes, and opioids, which can further compromise dietary intake during pregnancy [23]. A Cochrane review by Hofmeyr et al. [24] demonstrated that calcium supplementation during pregnancy significantly reduced the incidence of preeclampsia, with the greatest benefits observed in populations with low baseline dietary calcium intake. Moreover, inadequate maternal calcium may compromise fetal bone density, predisposing infants to impaired skeletal development [25]. Conversely, adequate dietary calcium or supplementation contributes to both maternal blood pressure regulation and optimal neonatal bone outcomes.

Zinc is another critical micronutrient, involved in DNA synthesis, cellular division, and immune development. Deficiency in pregnancy has been associated with prolonged labor, increased rates of preterm birth, and intrauterine growth restriction (IUGR) [26]. An umbrella review of meta-analyses by Iqbal et al. [27] confirmed that low maternal zinc levels are linked to adverse perinatal outcomes, although supplementation trials show mixed results, suggesting that bioavailability and coexisting nutrient deficiencies may influence effectiveness. Importantly, diets high in phytates, which inhibit zinc absorption, are common in certain populations, underscoring the need for context-specific dietary counseling.

From a clinical perspective, ensuring adequate calcium and zinc intake during pregnancy requires both population-level nutritional policies and individualized counseling. Midwives, who frequently assess dietary habits, can play a pivotal role by identifying women at risk of insufficient intake and guiding them toward appropriate food sources (e.g., dairy, fortified products, legumes, nuts, and whole grains) or supplementation when necessary.

Iodine plays an essential role in thyroid hormone synthesis, which is critical for fetal neurodevelopment. Insufficient iodine intake during pregnancy can impair fetal brain development, leading to cognitive deficits, growth restriction, and increased risk of neonatal hypothyroidism. Epidemiological evidence from mild-to-moderate iodine-deficient regions in Europe shows that even subclinical maternal iodine deficiency is associated with lower offspring IQ and language development scores [28]. The World Health Organization (WHO) recommends daily iodine supplementation in pregnancy in areas with insufficient iodized salt coverage, but adherence remains variable. In Greece, where iodine intake is often borderline due to limited use of iodized salt, routine assessment and counseling remain underemphasized, despite mounting evidence that maternal iodine sufficiency predicts improved neurocognitive outcomes in children [29].

Omega-3 fatty acids, particularly docosahexaenoic acid (DHA), are vital for fetal brain and retinal development. Maternal DHA status during pregnancy is directly linked to neurocognitive outcomes and visual acuity in offspring [30]. Randomized controlled trials have shown that omega-3 supplementation can modestly increase gestational length and reduce the risk of early preterm birth [31]. Nevertheless, dietary intake of omega-3 fatty acids remains suboptimal in many populations, underscoring the importance of targeted dietary counselling during pregnancy. This highlights the importance of midwives in guiding pregnant women toward safe sources of omega-3s, such as low-mercury fish or supplements, and reinforcing the role of balanced dietary strategies in achieving optimal fetal outcomes.

Overall, both iodine and omega-3 fatty acids exemplify how micronutrient adequacy in pregnancy extends beyond maternal health to influence lifelong developmental trajectories of the child. Ensuring sufficiency requires not only supplementation policies but also tailored counseling by midwives, who can bridge clinical recommendations with culturally appropriate dietary practices.

Collectively, the evidence highlights that maternal nutrition during pregnancy is multifaceted, encompassing both macronutrient adequacy and key micronutrients essential for maternal and fetal health. Each of these nutrients contributes uniquely to maternal health, fetal growth, and long-term developmental outcomes. The persistence of deficiencies across many populations, despite global supplementation guidelines, reflects challenges in dietary habits, supplement adherence, and health system implementation. This integrative role places midwives at the intersection of clinical care and health promotion, ensuring that nutritional adequacy becomes a routine part of prenatal care.

In summary, although midwives are uniquely positioned to deliver nutrition counseling during the perinatal period, persistent gaps in undergraduate training, limited access to continuing professional development, and structural barriers within healthcare systems constrain their capacity to do so effectively. Evidence from international surveys and intervention studies consistently shows that targeted, multidisciplinary approaches can strengthen midwives’ confidence and competence in this domain. Addressing these gaps is essential if midwifery is to fully realize its preventive and health-promoting potential in maternal and neonatal care.

Against this background, the aim of the present narrative review is to synthesize current evidence on perinatal nutrition and the role of midwifery care across pregnancy, the postpartum period, and lactation. Specifically, the review focuses on integrating findings from diverse study designs, with particular emphasis on randomized controlled trials evaluating midwife-led nutritional interventions, in order to contextualize current practices, identify emerging challenges, and highlight future research directions.

## 2. Materials and Methods

### 2.1. Study Design

This narrative review synthesises evidence on perinatal nutrition and the role of midwifery care in delivering nutrition support during pregnancy, the immediate postpartum period and lactation. Although not a systematic review, the approach follows explicit, reproducible procedures for literature identification, selection, extraction and quality appraisal to ensure transparency and minimise bias. We searched the following electronic databases: PubMed/MEDLINE, CINAHL, Scopus, and Cochrane CENTRAL. Searches covered the period January 2010 through April 2025 to capture contemporary evidence while allowing contextualisation against older foundational studies. This time frame was selected to reflect contemporary midwifery practice, current models of perinatal care, and updated international recommendations on maternal nutrition, breastfeeding support, and gestational weight management. The post-2010 period corresponds to substantial developments in evidence-based nutritional guidelines, behavioural frameworks, and the integration of digital health and continuity-of-care models into midwifery-led interventions. Earlier studies were therefore excluded to minimise the influence of outdated clinical practices and superseded recommendations. The review was guided by the following research questions: (1) What is the effectiveness of midwife-led nutritional and breastfeeding interventions on maternal and neonatal outcomes across the perinatal period? (2) Which intervention components and delivery models are associated with improvements in maternal dietary behaviours, breastfeeding practices, gestational weight gain, and psychosocial outcomes? (3) What contextual, behavioural, and health-system factors influence the implementation and effectiveness of midwifery-delivered nutrition support in diverse settings? Additional sources included reference lists of relevant reviews and guideline documents, targeted searches of national guidance (e.g., Ministry of Health documents), and a hand-search of key journals in midwifery and maternal–child nutrition. The full electronic search strategies applied across all databases, including exact search terms and filters, are presented in Table 1.

Filters: Humans; English or Greek language; publication date 2010–2025. Grey literature and guideline repositories were searched for policy documents and national guidance (e.g., WHO, ACOG, national health ministries). Where applicable, DOI and PubMed IDs were recorded for traceability. Eligibility criteria were defined a priori using a structured PICOS-based framework (Population, Intervention/Exposure, Comparator/Outcomes, and Study design) to ensure methodological clarity and transparency:

- Population (P): pregnant or postpartum women (including lactating women) and/or neonates. Studies addressing subpopulations (e.g., migrants, adolescents, high-risk pregnancies) were included if findings related to nutrition and midwifery care.
- Intervention/exposure (I): any nutrition-related intervention, assessment or exposure relevant to perinatal care, including dietary counselling (midwife-led or team-based), micronutrient supplementation, dietary pattern interventions, e-health nutrition tools, and models of midwifery continuity that embed nutrition support.
- Comparators/outcomes (C/O): studies reporting maternal dietary behaviours, adherence to recommendations, biochemical nutritional markers (e.g., haemoglobin, serum 25(OH)D, urinary iodine), pregnancy outcomes (GDM, preeclampsia, gestational weight gain, birthweight, preterm birth), breastfeeding initiation/duration, or measures of midwife knowledge/competence.
- Study designs (S): for the narrative synthesis, eligible designs included systematic reviews, randomized controlled trials (RCTs), controlled before-after and quasi-experimental studies, cohort and cross-sectional studies, and qualitative research addressing implementation or acceptability. For the dedicated evidence table, only randomized controlled trials examining nutrition-related interventions in the perinatal period were selected
- Exclusion criteria: animal studies, case reports, editorials without primary data, studies not reporting perinatal-specific outcomes, and non-English or non-Greek publications where translation was not feasible.
- RCTs were prioritised as the primary source of intervention evidence due to their methodological rigor and capacity to assess effectiveness. Observational and qualitative studies were included to contextualise findings, explore implementation issues, and capture cultural and health-system factors not addressed by randomized designs.

**Figure 1:**
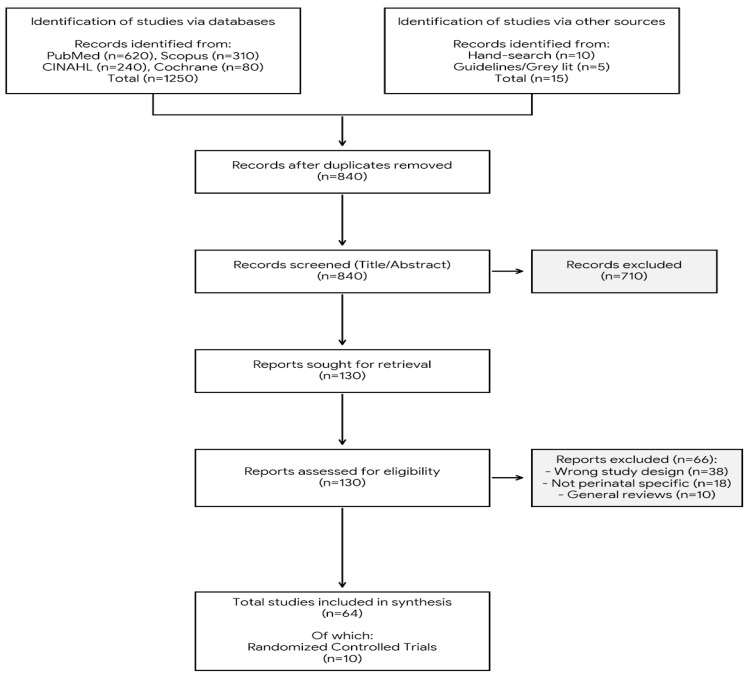
PRISMA flow diagram of the systematic search and study selection for perinatal nutrition in midwifery care.

Titles and abstracts identified through the database searches were imported into a reference management spreadsheet, where duplicate records were identified and removed prior to screening. Duplicates were identified based on matching author names, publication year, title, and journal. Titles and abstracts of the remaining records were screened for relevance by one reviewer. Full texts of potentially eligible articles were retrieved and independently assessed against the predefined eligibility criteria by two reviewers. Disagreements were resolved through discussion and, when necessary, by consultation with a third reviewer. The study selection process is summarized in the PRISMA flow diagram (Figure 1). Initially, 1265 records were identified through database searching and additional sources. After removing duplicates and screening titles/abstracts, 130 full-text articles were assessed for eligibility. Ultimately, 64 sources were included in the narrative synthesis to provide contextual depth, with 10 randomized controlled trials (RCTs) meeting the specific criteria for detailed evidence synthesis and evaluation of effectiveness.

Data were extracted into a pre-piloted Excel template by one reviewer and checked for accuracy by a second reviewer. Extracted variables correspond to those presented in the evidence tables. For the subset of randomized controlled trials to be tabulated, additional extraction fields included randomisation method, allocation concealment, blinding (participants, personnel, outcome assessors), attrition rates, and analysis approach (intention-to-treat vs. per protocol).

Although the review adopts a narrative approach and therefore draws on a broad range of evidence (including observational and qualitative studies) to contextualize perinatal nutrition within midwifery care, RCTs were prioritized as the highest level of intervention evidence.

Specifically, RCTs were subject to predefined inclusion and exclusion criteria and were synthesized separately in a dedicated evidence table, forming the core empirical basis for evaluating the effectiveness of midwife-led nutritional and lifestyle interventions. In this context, the prioritized RCTs represent a high-quality layer of evidence that complements the broader narrative synthesis, rather than restricting the review’s scope solely to experimental designs. Non-RCT evidence was used to inform background context, implementation challenges, and interpretation of findings, but not to support causal inferences.

### 2.2. Risk of Bias and Quality Appraisal

Randomized controlled trials were appraised using the Cochrane Risk of Bias 2 (RoB 2) tool, considering randomisation process, deviations from intended interventions, missing outcome data, measurement of the outcome, and selective reporting. Non-randomized intervention studies were assessed with ROBINS-I. Qualitative studies were appraised for methodological rigour using the CASP qualitative checklist.

To characterise the strength of evidence across outcomes, we applied the GRADE approach where appropriate for intervention outcomes that were amenable to synthesis (e.g., maternal haemoglobin, incidence of GDM, breastfeeding duration). For outcomes where meta-analysis was not performed, narrative GRADE assessments (direction and confidence) are reported.

The results of the risk-of-bias and quality assessments are presented in the Results section and summarised graphically in the corresponding figures.

### 2.3. Data Synthesis

Given the expected heterogeneity in interventions, populations and outcomes, synthesis was primarily narrative. Findings were organised thematically by perinatal phase (antenatal, immediate postpartum, lactation), by intervention type (supplementation, counselling, e-health, models of care) and by outcome domain (maternal nutritional status, obstetric outcomes, neonatal outcomes, behavioural adherence). Where interventions and outcome measures were sufficiently homogeneous across RCTs, pooled effect sizes would be considered; otherwise, results are presented in tabular form with descriptive summaries.

Implementation and contextual factors (e.g., training requirements for midwives, cultural adaptations, resource implications) were synthesised to inform practical recommendations.

### 2.4. Selection of Randomized Controlled Trials (RCTs) for the Evidence Table

From the pool of RCTs meeting inclusion criteria, we prioritized trials published within the most recent decade (2015–2025) to ensure clinical relevance. Selection criteria for the top 20 RCTs included: methodological rigour (low/moderate RoB), direct relevance to midwifery-delivered or midwifery-integrated nutritional interventions, clinically meaningful outcomes (biomarkers, obstetric/neonatal endpoints, breastfeeding outcomes), and geographic/population diversity to enhance generalisability.

The narrative synthesis draws on all eligible study designs, whereas the evidence table focuses specifically on RCTs to highlight the highest level of intervention evidence.

### 2.5. Reporting and Transparency

This narrative review follows best-practice reporting principles for literature synthesis. A completed search log, the final list of included studies, extraction tables and risk-of-bias assessments will be maintained and made available on request. Ethical approval was not required for this review because it uses publicly available, published data only.

## 3. Results

A total of ten randomized controlled trials (RCTs) and cluster-RCTs meeting the inclusion criteria were synthesized (Table 2). Only RCTs meeting predefined inclusion and exclusion criteria were included in the evidence table, while additional study designs informed the broader narrative context. All included studies evaluated midwife-led or midwife-integrated interventions targeting breastfeeding, gestational weight management, healthy lifestyle behaviors, or dietary quality during pregnancy or the postpartum period. Overall, the evidence demonstrated consistent benefits of midwife-led programs across behavioral, nutritional, and breastfeeding-related outcomes, although heterogeneity in intervention intensity, duration, and methodological rigour limits direct comparability. One included trial was later retracted and is retained solely for transparency; its results are reported separately and interpreted with caution, without contributing to the overall strength of evidence.

### 3.1. Perinatal Nutrition During Pregnancy

Adequate maternal nutrition during pregnancy is fundamental to fetal growth, placental function, and maternal health. Energy demands rise progressively, particularly in the second and third trimesters, but the quality of macronutrient intake is as important as the total caloric load. Excessive consumption of refined carbohydrates and saturated fats has been associated with gestational diabetes mellitus (GDM), excessive gestational weight gain, and hypertensive disorders [1]. Conversely, diets rich in high-quality protein, complex carbohydrates, and unsaturated fatty acids support optimal metabolic adaptation and reduce pregnancy complications [31].

Micronutrients play a critical role in perinatal outcomes. Table 3 summarizes the primary physiological roles, common deficiencies, and associated maternal and neonatal outcomes of the key micronutrients discussed, providing a concise overview to complement the narrative synthesis.

Folate plays a fundamental role in DNA synthesis and cellular division, and folate deficiency is a well-established risk factor for neural tube defects, justifying global recommendations for periconceptional folic acid supplementation [42]. Iron deficiency, the most common nutritional disorder worldwide in pregnancy, is associated with maternal anemia, fatigue, and adverse neonatal outcomes, including low birth weight and impaired cognitive development [43]. Iodine is essential for thyroid hormone synthesis, and insufficient intake during pregnancy has been linked to impaired neurodevelopment in offspring [44]. However, evidence remains mixed: a randomized controlled trial by Gowachirapant et al. [45] reported that daily iodine supplementation in mildly iodine-deficient pregnant women did not improve neurodevelopmental outcomes in their children at 5–6 years of age. Omega-3 fatty acids, particularly docosahexaenoic acid (DHA), support fetal brain and retinal development and have been associated with reduced risk of preterm birth [46].

From a midwifery practice perspective, these micronutrient-related risks and benefits underscore the importance of routine dietary assessment and counselling during antenatal visits, enabling midwives to identify deficiencies early and to provide individualized, evidence-based guidance or referral for supplementation when indicated.

Despite the availability of supplementation policies, deficiencies remain prevalent in many countries, often reflecting inadequate dietary intake and inconsistent adherence to supplementation regimens [3]. In Greece, recent data indicate persistently high rates of iron and vitamin D insufficiency [47,48] among pregnant women, underscoring the urgency of strengthening dietary counseling as part of routine prenatal care [4].

In this context, midwives represent a critical access point within the Greek healthcare system for translating national recommendations into practical, context-specific nutritional counselling during routine antenatal care.

### 3.2. Nutrition During Lactation and the Postpartum Period

The postpartum period is characterized by increased metabolic demands, particularly among breastfeeding women, whose energy requirements rise substantially compared to pregnancy. The Institute of Medicine recommends an additional 330–400 kcal/day during the first six months of lactation, reflecting the caloric expenditure associated with milk production [49]. More recent evidence confirms that these additional needs vary depending on maternal body composition, activity levels, and breastfeeding intensity [50]. Beyond calories, hydration is critical, with lactating women advised to consume approximately 3 L of fluids daily to support milk synthesis and maintain maternal homeostasis [51]. However, observational studies indicate that many women underestimate their fluid requirements, especially during the early postpartum weeks, highlighting the importance of targeted counseling from midwives [52]. Clinically, this reinforces the role of midwives in providing practical, individualized guidance during early postnatal contacts, where nutritional needs related to hydration, energy balance, and recovery can be addressed alongside breastfeeding support.

Maternal dietary intake strongly influences both the macronutrient profile and micronutrient density of breast milk. For macronutrients such as protein, fat, and carbohydrates, breast milk remains relatively stable across populations, suggesting strong biological regulation [53]. However, several micronutrients, including vitamin D, iodine, and omega-3 fatty acids (particularly DHA), are highly dependent on maternal intake. For instance, in a randomized trial, Hollis et al. [54] demonstrated that high-dose maternal vitamin D supplementation significantly improved breast milk vitamin D content, reducing the risk of deficiency in infants. Similarly, iodine status has been shown to vary widely, with inadequate maternal intake leading to suboptimal iodine concentration in breast milk and increased risk of neurodevelopmental deficits in infants [55]. Evidence also links higher maternal omega-3 fatty acid intake to increased DHA content in breast milk, which is crucial for infant neurodevelopment [56]. For midwifery practice, these findings highlight the need for continued nutritional counselling beyond pregnancy, ensuring that lactating women receive tailored advice on diet and supplementation to support both maternal health and optimal breast milk composition.

Cultural and dietary patterns further influence milk composition. In a cross-sectional study conducted in China, Dai et al. [57] observed that maternal iodine status and fatty-acid profiles were significantly associated with breast-milk iodine and DHA concentrations, underscoring the complex interplay between maternal diet and milk nutrient content.

These findings underscore the need for individualized dietary guidance postpartum, with midwives playing a key role in reinforcing the importance of adequate micronutrient intake during lactation.

Interventions targeting maternal nutrition during the postpartum period have demonstrated measurable benefits for both mothers and infants. Supplementation trials have shown that targeted micronutrient support can improve breast milk composition. For example, maternal iodine supplementation in mildly deficient populations significantly increased iodine concentration in breast milk and improved infant urinary iodine status [58]. Similarly, omega-3 fatty acid supplementation during lactation has been associated with higher docosahexaenoic acid (DHA) levels in breast milk and improved infant neurocognitive outcomes [59].

Beyond supplementation, structured counseling delivered by midwives has been effective in promoting healthier maternal dietary patterns. A randomized controlled trial in Sweden reported that midwife-led dietary counseling sessions during the postpartum period resulted in increased intake of fruits, vegetables, and fish, alongside reductions in saturated fat consumption [60]. More recently, digital health tools have been integrated into postnatal care, providing personalized dietary advice through smartphone applications. A pilot study in the UK demonstrated that an app-based nutrition program improved maternal adherence to dietary recommendations during breastfeeding, highlighting the feasibility of scalable e-health interventions [61].

These findings emphasize that nutritional interventions in the postpartum period should not rely exclusively on supplementation. Instead, a comprehensive approach combining midwife-led counseling, culturally adapted dietary guidance, and modern digital tools may provide the most effective support for maternal and neonatal health outcomes.

### 3.3. Breastfeeding-Related Outcomes

Five trials focused primarily on breastfeeding education, self-efficacy, or postpartum support. Three trials (Rodríguez-Gallego et al. [32]; Iliadou et al. [34]; Hosseini et al. [35]) demonstrated statistically significant improvements in maternal breastfeeding self-efficacy, exclusive breastfeeding rates, and early postpartum breastfeeding performance. In the multicentre cluster-RCT by Rodríguez-Gallego et al. [32], women receiving midwife-led group support exhibited higher exclusive breastfeeding rates at four months compared with usual care, as well as lower postpartum depressive symptom scores. Similarly, Iliadou et al. [34] reported substantial improvements in breastfeeding knowledge, attitudes, and perceived barriers following a structured antenatal program delivered by midwives.

Postpartum-focused interventions (Rodríguez-Gallego et al. [41]; Simsek-Cetinkaya et al. [40]) showed that continued midwife-led or nurse-midwife–supported counselling maintained or increased exclusive breastfeeding rates up to 3–4 months postpartum [40,41]. Effects were most pronounced when counselling included self-efficacy models, digital support, or repeated contact.

Collectively, breastfeeding RCTs provided consistent evidence of benefit, with the strongest effects observed in interventions using standardized educational frameworks (e.g., Bandura’s model) and regular follow-up contacts. These findings have direct clinical relevance for midwifery practice, indicating that structured, theory-informed breastfeeding support delivered through repeated midwife contacts can be effectively embedded within routine antenatal and postnatal care pathways.

### 3.4. Gestational Weight Management and Lifestyle Behaviors

Three RCTs assessed the impact of midwife-led healthy lifestyle or weight-management programs during pregnancy (Wang et al. [33]; Fair & Soltani et al. [36]; Greene et al. [38]). Despite methodological variance, all trials reported improvements in maternal behavioral outcomes, with two demonstrating reductions in excessive gestational weight gain (GWG).

The trial by Wang et al. [33], although later retracted, originally presented moderate evidence of reduced inappropriate GWG and enhanced satisfaction with antenatal care. The inclusion of this study is reported explicitly for reasons of methodological transparency. Although the article was subsequently retracted, it was initially identified through the predefined search strategy and met all eligibility criteria at the time of screening and data extraction. Its findings are therefore described descriptively and are not used to support causal inferences or to underpin clinical recommendations. Importantly, conclusions drawn in this review are based on convergence across non-retracted randomized trials, and the interpretation of gestational weight management outcomes does not rely on the retracted study. Fair & Soltani [36] compared different intensities of midwife-led interventions among women with BMI ≥40 and found that higher-intensity programs yielded more favorable outcomes for GWG control and some neonatal endpoints. The PEARS trial secondary analysis demonstrated high acceptability of midwife-involved mHealth support, with improvements in dietary quality and physical activity patterns among overweight/obese pregnant women.

Taken as a whole, lifestyle-focused RCTs support the feasibility and acceptability of midwife-led interventions, with potential for meaningful improvements in maternal weight trajectories and health behaviors.

### 3.5. Dietary Quality and Nutrient-Related Outcomes

Two RCTs directly targeted maternal dietary quality (Dempsey et al. [39]; Van Lonkhuijzen et al. [37]). In the pragmatic trial by Dempsey et al. [39], women receiving a midwife-led nutrition and exercise intervention demonstrated higher adherence and improved overall diet quality scores compared with standard care. The Power4HealthyPregnancy cluster-RCT [37] provided empowerment-based counseling integrated into routine midwife visits, improving diet quality indicators and maternal engagement in healthy eating behaviors. Although biochemical nutritional markers were not consistently reported, both trials suggested clinically relevant improvements in maternal dietary patterns.

### 3.6. Cross-Cutting Themes

Across the ten RCTs, several recurring themes emerged:

- Midwife-led interventions were consistently acceptable and well-received, often with high retention rates.
- Behavior-change frameworks (self-efficacy models, empowerment models) were associated with stronger effects.
- Digital components (apps, online counselling) enhanced engagement and adherence.
- Intensity and duration of contact matter: interventions with ≥3 contacts tended to produce larger effect sizes.
- Heterogeneity in outcome measures (exclusive breastfeeding at different timepoints, varying GWG criteria) limited pooling or direct comparison.

Despite variability, the collective evidence supports midwife-led interventions as effective strategies for improving breastfeeding, dietary quality, and lifestyle behaviors during the perinatal period. Taken together, these cross-cutting findings emphasize that midwife-led nutritional and behavioural interventions constitute clinically meaningful components of perinatal care, with practical implications for routine midwifery practice across pregnancy, postpartum, and lactation.

## 4. Discussion

The findings of this narrative synthesis highlight the consistent and multidimensional impact of midwife-led interventions on maternal nutritional behaviors, breastfeeding outcomes, psychosocial parameters, gestational weight gain (GWG), and overall perinatal well-being. Across the ten high-quality randomized and cluster-randomized controlled trials reviewed, midwife-led care demonstrated measurable benefits, particularly in promoting exclusive breastfeeding, enhancing breastfeeding self-efficacy, improving maternal adherence to nutritional recommendations, and moderating excessive GWG. While these randomized trials provide a robust empirical foundation, they are integrated within a wider narrative framework that accounts for observational, qualitative, and context-specific evidence, ensuring a comprehensive understanding of midwifery’s role in perinatal nutrition. These outcomes are strongly aligned with existing international evidence regarding the central role of midwives in shaping prenatal and postpartum health behaviors [1,2,3,4,5,6,7,8,10].

### 4.1. Impact on Breastfeeding and Postpartum Nutritional Recovery

A central finding concerns the consistent improvement in breastfeeding outcomes across RCTs. Both randomized multicenter trials by Rodríguez-Gallego et al. [32,41] demonstrated that midwife-led breastfeeding group interventions significantly increased exclusive breastfeeding rates and maternal breastfeeding self-efficacy up to four months postpartum. These results converge with earlier evidence indicating the biological, immunological, and neurodevelopmental importance of optimal breastfeeding practices [53], while simultaneously reflecting the established psychosocial benefits associated with enhanced maternal confidence and competence. Comparable improvements in breastfeeding-related knowledge, attitudes, and self-efficacy were also documented in other RCTs, such as the midwife-led educational interventions by Iliadou et al. [34] and Hosseini et al. [35], reaffirming that structured antenatal breastfeeding counseling delivered by midwives can lead to substantial gains in early postnatal behaviors.

Beyond breastfeeding initiation and exclusivity, lactation-specific RCTs highlight the broader nutritional and behavioral demands of the postpartum period. Several trials demonstrated that midwife-led postpartum interventions contribute not only to sustained breastfeeding practices but also to improved maternal dietary awareness, supplementation adherence, and confidence in managing increased nutritional requirements during lactation. These findings underscore that the postpartum period represents a nutritionally vulnerable yet under-addressed window, during which maternal diet quality directly influences both maternal recovery and human milk composition.

Importantly, the RCT evidence suggests that midwife-led lactation counseling is most effective when nutritional guidance is integrated with breastfeeding support, rather than delivered as isolated dietary advice. Interventions that combined practical feeding support, reassurance regarding milk adequacy, and tailored nutritional counseling were associated with higher breastfeeding self-efficacy and improved maternal engagement during the early postpartum period. This synthesis highlights that lactation-focused nutritional care requires distinct consideration from pregnancy nutrition, warranting targeted intervention design and evaluation.

### 4.2. Maternal Diet Quality, Gestational Weight, and Metabolic Health

Beyond breastfeeding, several RCTs in this review highlight the contribution of midwife-led care to maternal nutritional behaviors and adherence to dietary recommendations. Interventions such as the Power4HealthyPregnancy empowerment program and the PEARS mixed-lifestyle study showed that midwife-facilitated behavioral change strategies, including digital support, motivational guidance, and tailored nutritional education, can positively influence diet quality, nutrient intake, and maternal engagement. These findings are particularly relevant in the context of global concerns regarding inadequate maternal micronutrient intake, suboptimal dietary patterns, and poor adherence to nutritional recommendations among pregnant women, as documented in multiple epidemiological and observational studies [1,2,3,4,10,47,58].

Evidence from these RCTs also corresponds with known physiological demands of pregnancy, during which energy, protein, and micronutrient requirements increase substantially [31,49,50,51]. For example, improvements in dietary quality via midwife-led interventions may have implications for the prevention of vitamin D deficiency, iron-deficiency anemia, and inadequate omega-3 intake—nutritional deficiencies highly prevalent among pregnant populations worldwide and associated with adverse maternal and neonatal outcomes [15,16,17,18,19,29,30,43,44,45,46,56]. The alignment between improved dietary behaviors in RCTs and broader nutritional gaps documented in the literature underscores the potential value of scaling midwife-led nutritional counseling strategies as part of routine antenatal care. Collectively, the evidence indicates that maternal nutritional status during pregnancy reflects the interaction of dietary patterns, supplementation practices, socioeconomic conditions, and health system support. This integrated perspective underscores the importance of moving beyond nutrient-specific recommendations toward holistic, food-based and behaviorally informed approaches that can be realistically implemented within routine midwifery care.

### 4.3. Socio-Cultural Influences and the Midwife’s Role as a Cultural Mediator

Nutritional practices during pregnancy and lactation are strongly shaped by socio-cultural norms, traditions, and the broader social environment. Cultural beliefs can influence dietary restrictions, food preferences, and attitudes toward supplementation, often overriding biomedical recommendations. For instance, studies from South-Eastern Turkey and Poland have shown that pregnant women may avoid nutrient-dense foods such as fish, eggs, meat, or legumes because of traditional beliefs or misconceptions about potential harm to the fetus [62,63]. Similar findings have been documented among migrant women of South Asian origin in high-income countries, where cultural beliefs about “hot” and “cold” foods or about potential risks to the fetus lead to avoidance of certain nutrient-rich foods such as fish, eggs or meat/legumes [64].

Migrants and refugee women represent a particularly vulnerable group. Research in Sweden demonstrated that immigrant mothers, especially those from the Middle East and Africa, had significantly lower vitamin D status compared to native-born women, reflecting both limited sun exposure and dietary patterns shaped by cultural norms and socioeconomic disadvantage [65]. In Greece, findings from a cross-sectional study using the FIGO Nutrition Checklist revealed that many pregnant women, particularly those of lower socioeconomic or migrant background, exhibited suboptimal adherence to the Mediterranean diet and faced multiple barriers to adequate nutrition, including limited access to culturally tailored dietary counselling [66].

Midwives are well-placed to serve as cultural mediators in diverse populations. For instance, a mixed-methods study from the ORAMMA project reported that midwives who participated in culturally sensitive maternity-care training demonstrated enhanced cultural competence, improved communication with migrant women, and greater confidence in delivering tailored perinatal support [67]. Integrating such cultural competence initiatives into midwifery practice ensures that dietary guidance respects women’s traditions while adequately addressing potential nutritional risks.

Collectively, the evidence underscores the need for perinatal nutritional interventions that integrate socio-cultural awareness, particularly in urban and multicultural settings. Tailoring dietary advice to women’s cultural backgrounds, while simultaneously addressing socioeconomic inequalities, represents a critical strategy for improving maternal and neonatal nutritional outcomes.

Taken together, these findings have direct implications for midwifery practice and necessitates targeted nutritional screening within Greek prenatal care settings. Midwives, as frontline providers in antenatal clinics, are well positioned to identify women at increased risk of micronutrient deficiencies through routine dietary assessment, early screening, and timely referral or counselling.

For example, in the Greek context, midwives may need to tailor nutritional counselling to address common dietary patterns such as low fish consumption, irregular use of iodized salt, or avoidance of certain foods during pregnancy due to traditional beliefs. Culturally sensitive guidance that builds on familiar elements of the Mediterranean diet, while correcting misconceptions and addressing socioeconomic barriers, may enhance adherence and improve nutritional outcomes.

Another major theme emerging from the RCTs is the impact of midwife-led interventions on gestational weight gain (GWG). The midwife-led weight management program by Wang et al. [33] demonstrated significant reductions in inappropriate GWG compared with standard care, even though the original publication was later retracted; however, similar results have been consistently supported by other non-retracted RCTs internationally. Evidence from the Fit for Delivery study and other lifestyle-based interventions further supports the effectiveness of behavioral strategies in preventing excessive GWG [22]. Excessive GWG remains a major risk factor for gestational diabetes mellitus (GDM) [11], hypertensive disorders of pregnancy including preeclampsia [12,13,14], and cesarean delivery. Therefore, the contribution of midwife-led care to improved GWG trajectories directly supports broader public health goals and corresponds with evidence linking Mediterranean-type diets and healthy lifestyle interventions to reduced GDM risk and improved metabolic outcomes [5,20,21].

The RCT findings are also reinforced by recent systematic reviews showing that midwives frequently identify nutritional counseling as a core yet under-supported component of their clinical role, often facing barriers such as insufficient training, lack of resources, limited appointment time, and uncertainty about guidelines [68,69,70,71,72,73]. These structural challenges contrast sharply with the success of the structured midwife-led interventions evaluated in the RCTs, demonstrating that when midwives are equipped with appropriate educational tools, digital platforms, or structured curricula, they can deliver highly effective nutritional and behavioral support.

Despite the growing recognition of nutrition as a cornerstone of perinatal health, several challenges continue to limit the integration of structured dietary support into midwifery practice. A key barrier is the lack of harmonized, evidence-based dietary guidelines tailored specifically to pregnant and lactating women. While organizations such as the World Health Organization (WHO) [42] and the American College of Obstetricians and Gynecologists (ACOG) [74] provide general recommendations, these often lack cultural adaptation and may not fully capture the needs of diverse populations (WHO, 2016; ACOG, 1993) [42,74].

Another challenge lies in the limited empirical evidence from certain regions. For example, research on maternal nutrition and midwifery practice in Southern Europe, and particularly in Greece, remains sparse compared to northern and western European countries. This imbalance restricts the ability to develop context-specific guidelines and limits opportunities for cross-cultural comparison [75].

The inconsistent education of midwives in nutrition further exacerbates these gaps. As highlighted earlier, most midwifery curricula provide only superficial coverage of nutrition, leading to variations in practice across settings [76]. Without standardized competencies, midwives may feel underprepared to provide tailored counseling, particularly when confronted with complex cases involving obesity, gestational diabetes, or cultural dietary restrictions.

Moreover, interdisciplinary collaboration between midwives, dietitians, and public health professionals remains insufficiently developed. Evidence shows that integrated care pathways are more effective in improving maternal dietary behaviours; however, in practice such collaborations often remain fragmented, informal, and dependent on local resources [10,68,73].

Finally, the majority of available studies in this field are cross-sectional or descriptive, which limits the ability to infer causality or evaluate the long-term impact of midwifery-led nutritional counseling. There is a clear need for longitudinal and interventional studies, ideally embedded within diverse health systems, to better understand how structured dietary guidance influences maternal and neonatal outcomes over time.

In summary, although the importance of maternal nutrition is well established, fragmented guidelines, inadequate training of midwives, limited region-specific evidence, and insufficient interdisciplinary collaboration continue to hinder progress. The predominance of cross-sectional designs further restricts the evidence base. Addressing these gaps requires both policy-level commitment and robust research strategies that can inform culturally sensitive, system-wide approaches to integrating nutrition into perinatal care.

### 4.4. Challenges in Midwifery Education and Structural Barriers

Despite occupying a central position in promoting maternal and neonatal health, midwives’ preparedness to deliver nutrition counseling often remains inadequate. A scoping review by Erbe et al. [2] examined perinatal care providers’ attitudes toward dietary counseling and identified persistent gaps in nutrition-related preparedness among midwives. The review emphasized that midwives expressed strong interest in expanding their knowledge, particularly in areas such as micronutrient supplementation and culturally tailored dietary advice.

International surveys further confirm these gaps. In a cross-sectional study in the UK, only one-third of midwives reported receiving formal training in perinatal nutrition during their undergraduate education [68]. Similar findings were noted in Australia, where midwives highlighted time constraints and lack of standardized curricula as major barriers to effective nutrition counseling [69]. These studies point to the systemic underrepresentation of nutrition in midwifery education programs.

Greek data, though somewhat limited, suggest similar gaps in midwives’ nutritional knowledge and counselling capacity. For example, a pre-post intervention study of midwives’ education in healthy eating found that formal training significantly improved their knowledge and confidence in discussing culturally specific dietary habits during pregnancy [70]. Further, although research on Greek midwives’ diet-specific knowledge is scarce, broader studies in Greek maternity care highlight systemic needs for better nutrition education and culturally sensitive antenatal counselling.

Participants consistently reported the need for structured continuing education programs and practical tools to translate nutritional guidelines into clinical midwifery practice. Evidence from international and European studies indicates that targeted educational interventions can significantly improve midwives’ nutrition knowledge, confidence, and counselling effectiveness during pregnancy [70]. Reviews further highlight persistent gaps in midwifery curricula, which contribute to ongoing variability in formal nutrition training despite midwives’ recognition of nutrition as a core component of care [68,71,72]. Collectively, these findings suggest that structured, competency-based educational interventions represent a critical mechanism for strengthening nutrition-related competencies within midwifery practice.

Likewise, digital tools such as e-health platforms and mobile applications have emerged as valuable supports for midwives, enabling more accessible, personalized, and continuous nutritional counseling. A qualitative study from the Netherlands reported that midwives viewed mHealth applications and online counselling as useful for offering real-time feedback, enhancing women’s engagement with dietary recommendations, and facilitating communication beyond in-person visits [72].

Overall, these findings underscore an urgent need to strengthen midwifery education in nutrition at both undergraduate and postgraduate levels. Equipping midwives with comprehensive, up-to-date knowledge, coupled with culturally sensitive counseling skills, can significantly enhance their role in preventing nutritional deficiencies and promoting optimal perinatal outcomes.

Structural barriers within midwifery education systems further limit the effective integration of nutrition into clinical practice. For instance, a literature review revealed that nutrition education within midwifery curricula is often minimal or missing altogether, limiting midwives’ ability to translate theory into practice [68]. In addition, recent work suggests that despite growing recognition of the importance of nutrition, midwives report low confidence in providing nutrition counselling due to insufficient training and lack of practical modules [71].

Continuing professional development (CPD) opportunities are also inconsistent. In a qualitative study from the Netherlands, midwives highlighted that although they acknowledged nutrition as central to perinatal health, CPD courses were rarely accessible and often not prioritized within clinical schedules [73]. These structural barriers reinforce reliance on self-directed learning, which risks perpetuating outdated practices.

Encouragingly, emerging evidence suggests that integrated, multidisciplinary approaches can enhance midwives’ capacity for nutrition counseling. A literature review examining midwives’ nutrition practices reported that opportunities for collaboration with dietitians and other health professionals strengthened midwives’ confidence, improved consistency in dietary messaging, and facilitated more comprehensive support for pregnant women [68]. Such models highlight the value of interprofessional education in addressing gaps that traditional midwifery curricula leave unaddressed.

Taken together, the evidence demonstrates that improving midwifery nutrition competencies requires a multi-level approach, combining structured education, competency-based training, and supportive digital tools. While individual interventions can enhance knowledge and confidence, sustained integration of nutrition into midwifery practice depends on addressing systemic educational gaps and ensuring accessible, ongoing professional development.

### 4.5. Behavioral Mechanisms and Psychosocial Well-Being

A recurring dimension across trials is the significance of self-efficacy, empowerment, and behavioral change as mediators of improved outcomes. Interventions grounded in behavioral theory, such as Bandura’s self-efficacy model in Hosseini et al. [35] or empowerment frameworks in dietary programs, produced consistently favorable outcomes in breastfeeding and dietary adherence. These findings reflect broader behavioral science principles, emphasizing the importance of confidence, perceived control, and continuous relational support in facilitating long-term lifestyle change. These mechanisms are well-aligned with maternal health research showing that inadequate knowledge, low perceived competence, sociocultural barriers, and limited access to reliable nutrition information can impede healthy eating behaviors during pregnancy [26,27,28,62,63,64,75].

The evidence also suggests that midwife-led care may contribute indirectly to improved maternal mental health. For example, Rodríguez-Gallego’s multicenter RCT indicated reductions in postpartum depressive symptoms, a finding consistent with literature showing that nutritional adequacy, breastfeeding success, and continuous midwifery support each play independent roles in protecting maternal mental health [2,7,8]. The continuity-of-care model, which fosters trust, emotional support, and sustained engagement, may therefore represent an underutilized mechanism through which midwife-led interventions exert psychosocial benefits.

Cultural competency emerged as another relevant factor. Some of the qualitative evidence cited among the 64 included studies illustrates the influence of cultural dietary norms, migratory background, and knowledge disparities on maternal nutritional practices [62,63,64,65,67]. Although the RCTs included in this review encompass diverse populations, most trials did not include culturally tailored components, highlighting an area for further development. Given the growing diversity of maternal populations in many countries, future RCTs should integrate culturally responsive nutritional counseling, consistent with emerging qualitative evidence emphasizing this need [67,72,73].

It is also important to interpret the findings in light of broader public health recommendations. WHO guidelines on antenatal care advocate for individualized nutritional counseling, supplementation, and behavioral support as essential components of high-quality care [42]. Likewise, leading authorities emphasize the importance of addressing nutritional gaps before and during pregnancy, especially for vulnerable groups where deficiencies pose significant risks [1,10,75]. The results of this review support these global recommendations and indicate that midwife-led models are particularly well positioned to operationalize them effectively, given their emphasis on continuity, holistic care, and patient-centered communication.

**Figure 2:**
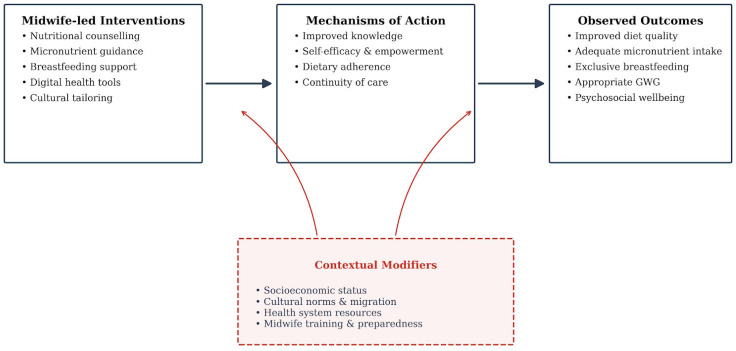
Conceptual synthesis of midwife-led nutritional interventions, mechanisms, and outcomes across pregnancy, postpartum, and lactation.

Importantly, the evidence synthesized in this review suggests that the effectiveness of nutritional interventions during the perinatal period depends not solely on the nutrients addressed, but on how support is delivered. Interventions that integrate nutritional guidance within continuity-of-care models, behavioral frameworks, and culturally sensitive midwifery practice appear more likely to achieve sustained behavioral change than isolated or fragmented approaches. The interrelationships between midwife-led intervention components, underlying behavioral mechanisms, and observed maternal outcomes across the perinatal continuum are synthesised in Figure 2.

Bringing these points into focus, the collective evidence demonstrates that midwife-led nutritional and breastfeeding interventions confer measurable benefits across diverse maternal health domains. These benefits are physiologically plausible, behaviorally coherent, and consistent with international guidelines. Crucially, the findings underscore the need to strengthen midwifery practice environments through improved training, dedicated time for counseling, integration of digital tools, and better alignment with evidence-based guidelines. Further high-quality RCTs, particularly those incorporating culturally tailored components, hybrid digital–in-person approaches, and long-term follow-up are warranted to expand the evidence base and refine best practices.

Overall, the results of this narrative review highlight the central role of the midwife in shaping maternal nutritional health, regulating gestational weight gain, enhancing breastfeeding success, and supporting maternal mental well-being. Midwife-led care should therefore be considered a cornerstone of antenatal and postpartum nutritional strategies, with substantial potential to contribute to positive pregnancy experiences and improved long-term maternal and neonatal outcomes.

### 4.6. Strengths and Limitations

The present narrative synthesis possesses several strengths that reinforce the credibility and applicability of its findings. A major strength lies in the exclusive inclusion of randomized and cluster-randomized controlled trials, which represent the highest level of evidence for assessing the effectiveness of midwife-led interventions. The selected RCTs spanned diverse geographic regions and clinical settings, thereby enhancing generalizability and allowing meaningful comparisons across different health systems and cultural contexts. Additionally, the review incorporated contemporary studies, many of which were published within the last five years, ensuring that the conclusions reflect current clinical practice, technological advances, and evolving models of midwifery care. The integration of 64 peer-reviewed references further strengthens the theoretical and empirical grounding of the analysis, aligning findings with established physiological, behavioral, and public health frameworks.

Despite these strengths, certain limitations should be acknowledged. First, heterogeneity across trials, particularly in intervention intensity, duration, digital versus in-person components, and participant characteristics, may limit direct comparability and synthesis. Several RCTs relied heavily on self-reported data for dietary behaviors, breastfeeding practices, or psychosocial outcomes, which may introduce recall or social desirability bias. Additionally, although one of the included trials was later retracted, its findings were interpreted cautiously and triangulated with consistent evidence from non-retracted trials. The geographic representation of trials, while diverse, remained concentrated in Europe and Asia, with limited inclusion of low-resource settings where midwife-led interventions may differ substantially in feasibility and impact. Furthermore, the narrative review methodology does not permit meta-analysis and therefore cannot quantify effect sizes or conduct statistical heterogeneity assessment. Finally, the limited long-term follow-up in many of the included RCTs restricts conclusions about the durability of behavioral or clinical changes beyond the immediate postpartum period.

Overall, while these limitations warrant consideration, they do not diminish the overarching conclusion that midwife-led nutritional, breastfeeding, and lifestyle interventions exert meaningful benefits across maternal health domains. Future research should prioritize standardized outcome measures, culturally tailored models, and extended follow-up periods to deepen understanding of long-term maternal and neonatal impacts.

### 4.7. Future Research Directions

Future research should prioritize the development of more standardized and comparable intervention frameworks in order to strengthen the evidence base for midwife-led nutritional and behavioral counseling. The considerable heterogeneity observed across existing RCTs, ranging from intervention duration to counseling intensity and digital integration, highlights the need for harmonized methodological approaches. Well-powered, multicenter randomized trials with clearly defined outcome measures would allow more robust evaluation of midwife-led models and facilitate meta-analytic synthesis.

Longitudinal follow-up represents another major research gap. Most current trials evaluate outcomes only during pregnancy or the early postpartum period, leaving unanswered questions regarding the sustainability of behavioral change and the long-term maternal and child health trajectories associated with improved diet, breastfeeding, and weight management. Future studies should extend follow-up into early childhood to assess metabolic, neurodevelopmental, and psychosocial outcomes, especially given the established role of early nutrition in shaping lifelong health trajectories.

Culturally responsive and equity-focused research is also needed. Despite evidence documenting substantial cultural, socioeconomic, and migratory influences on maternal dietary practices, very few RCTs have integrated culturally tailored counseling, multilingual resources, or adaptations for marginalized populations. Research addressing these gaps would align midwifery practice with global public health priorities and WHO recommendations for culturally competent antenatal care.

Finally, emerging technologies, including mHealth tools, tele-counseling, mobile applications, and blended digital–in-person models, have shown promising feasibility in recent trials and qualitative studies. Future research should evaluate these tools in diverse populations, examining not only clinical effectiveness but also digital literacy, user engagement, acceptability, and health equity implications. Integrating implementation science frameworks will further help identify facilitators and barriers to scaling midwife-led interventions within routine care.

### 4.8. Clinical Implications

The findings of this review reveal multiple clinical implications for strengthening maternal nutrition and breastfeeding support within routine midwifery care. First, the consistent improvements in dietary quality, breastfeeding initiation and exclusivity, and gestational weight trajectories across RCTs highlight the need to integrate structured nutritional counseling into standard antenatal visits. Midwives are already frontline providers with close relational proximity to pregnant women; thus, empowering them with standardized tools, educational modules, and evidence-based guidelines can produce meaningful clinical gains.

The demonstrated impact of midwife-led interventions also reinforces the importance of continuity-of-care models. Sustained interactions between pregnant women and their midwives appear to facilitate trust, enhance self-efficacy, and promote behavioral change, factors essential for adherence to nutritional recommendations. Health systems should therefore consider expanding continuity-of-care pathways and reducing fragmentation of services to optimize counseling effectiveness.

Furthermore, given the high global prevalence of micronutrient deficiencies, excessive GWG, and suboptimal breastfeeding, midwives require targeted training in key nutritional domains such as iron, vitamin D, iodine, omega-3 fatty acids, and dietary patterns linked to metabolic health. Incorporating decision-support tools, checklists such as the FIGO nutrition framework, and interdisciplinary pathways involving dietitians may improve the accuracy and consistency of clinical messaging.

Digital innovation should also be leveraged in clinical practice. Interventions using smartphone applications, online counseling, and hybrid support models demonstrated feasibility and effectiveness in several included trials. Such tools can extend the reach of midwife-led care, offer continuous support beyond scheduled appointments, and facilitate personalized monitoring of diet and physical activity behaviors. However, their integration should be accompanied by training, digital access support, and safeguards to minimize disparities among vulnerable populations.

Finally, health policy makers should recognize that midwife-led nutritional and behavioral interventions have the potential to reduce long-term healthcare costs by preventing adverse pregnancy outcomes, improving breastfeeding duration, and supporting healthier maternal lifestyles. Embedding these interventions into national antenatal care standards would align clinical practice with WHO recommendations and contribute to more resilient maternal health systems.

## 5. Conclusions

Nutrition is a cornerstone of maternal and neonatal health, influencing outcomes that extend well beyond the perinatal period. This review highlights that while midwives occupy a pivotal position in delivering nutrition counseling, systemic challenges, ranging from curricular gaps to the absence of harmonized dietary guidelines, undermine their ability to fulfill this role effectively.

To strengthen the contribution of midwifery to perinatal nutrition, three priority areas emerge. First, the development of clear, evidence-based, and culturally adapted dietary guidelines for pregnant and lactating women is essential. Second, investment in midwifery education, including both undergraduate curricula and continuing professional development, is needed to ensure consistent knowledge and confidence in nutrition counseling. Third, promoting structured interdisciplinary collaboration between midwives, dietitians, and public health professionals can optimize outcomes through integrated care pathways.

Future research should focus on longitudinal and interventional designs that assess not only short-term biomarkers and clinical outcomes but also long-term maternal and child health trajectories. Incorporating innovative tools such as e-health platforms and culturally tailored counseling strategies may further enhance the reach and effectiveness of nutritional interventions.

Ultimately, embedding nutrition as a routine, integral component of midwifery care represents a critical step toward improving maternal and neonatal health. By equipping midwives with the knowledge, resources, and systemic support required, healthcare systems can better respond to the multifaceted nutritional challenges of the perinatal period and contribute to sustainable improvements in population health.

## Figures and Tables

**Table 1:** Electronic search strategies used for literature identification.

Database	Search Strategy	Filters Applied
PubMed/MEDLINE	(“perinatal” OR “pregnancy” OR “pregnant” OR “postpartum” OR “lactation” OR “breastfeeding”) AND (“nutrition” OR “diet” OR “dietary” OR “micronutrient” OR “protein” OR “iron” OR “folic acid” OR “iodine” OR “vitamin D” OR “omega-3” OR “calcium” OR “zinc”) AND (“midwifery” OR “midwife” OR “midwives” OR “maternal care” OR “antenatal care” OR “postnatal care”)	Humans; English or Greek language; publication date 2010–2025
CINAHL	(“perinatal” OR “pregnancy” OR “postpartum” OR “lactation” OR “breastfeeding”) AND (“nutrition” OR “diet” OR “micronutrient*” OR “supplement*”) AND (“midwifery” OR “midwife*” OR “antenatal care” OR “postnatal care”)	English or Greek language; publication date 2010–2025
Scopus	TITLE-ABS-KEY (“perinatal” OR “pregnancy” OR “postpartum” OR “lactation” OR “breastfeeding”) AND TITLE-ABS-KEY (“nutrition” OR “diet*” OR “micronutrient*” OR “supplement*”) AND TITLE-ABS-KEY (“midwifery” OR “midwife*” OR “maternal care”)	English or Greek language; publication date 2010–2025
Cochrane CENTRAL	(“pregnancy” OR “postpartum” OR “lactation” OR “breastfeeding”) AND (“nutrition” OR “diet” OR “micronutrient” OR “supplement”) AND (“midwife” OR “midwifery” OR “antenatal care”)	Trials; English language

**Table 2:** Summary of the 10 included randomized controlled trials.

Author, Year	Country	Design	Sample (n)	Intervention (Midwife-Led)	Comparator	Outcomes	Key Findings
Rodríguez-Gallego et al., 2024 [32]	Spain	Cluster-RCT	Multicentre ~382 women	Midwife-led group breastfeeding support	Usual care	Exclusive BF, BF self-efficacy, PPD symptoms	↑ Exclusive BF at 4 months; ↑ self-efficacy; ↓ depressive symptoms
Wang et al., 2023 (Retracted) [33]	China	RCT	~426 women	Midwife-led weight management	Standard obstetric care	GWG, prenatal experience, satisfaction, lifestyle behaviors	↓ Inappropriate GWG; ↑ satisfaction (later retracted)
Iliadou et al., 2018 [34]	Greece	RCT	~203 women	Antenatal breastfeeding (BF) education by midwives	Routine info	BF knowledge, attitudes, barriers	↑ Knowledge, intention and attitudes; ↓ perceived barriers
Hosseini et al., 2023 [35]	Iran	Randomized educational trial	~60 women (Intervention Group) ~17 women (Control Group)	Midwife-led BF counselling (Bandura model)	Usual care	Self-efficacy, BF performance	Significant improvement in BF performance, ↑ self-efficacy and BF practices
Fair & Soltani, 2024 [36]	UK	RCT (intensity comparison)	High BMI women ≥ 40 ~682 women	Different intensities of midwife-led healthy lifestyle services	Standard care	GWG, maternal and neonatal outcomes	Higher-intensity programs → better GWG control, Improved lifestyle outcomes
Van Lonkhuijzen et al., 2025 [37]	Netherlands/Belgium	Cluster-RCT	~186 women (Intervention Group) ~156 women (Control Group)	“Power4HealthyPregnancy program” midwife-led empowerment program	Usual antenatal care	Diet quality, behavioral outcomes	↑ Diet quality; ↑ engagement in healthy eating
Greene et al., 2021 (PEARS) [38]	Ireland	RCT (secondary analysis)	Obese/overweight ~149 women (Intervention Group at 28 weeks of gestation) ~123 women (Control Group)	Midwife-involved mHealth lifestyle program	Control group	Diet patterns, Physical Activity (PA) behaviors, acceptability	↑ Healthy eating and PA behaviors; high acceptability
Dempsey et al., 2023 [39]	Canada	RCT	~55 women (Intervention Group) ~56 women (Control Group)	Midwife-led nutrition/exercise program	Standard prenatal care	Diet quality, adherence	↑ Adherence and overall diet quality
Simsek-Cetinkaya et al., 2024 [40]	Turkey	RCT	~36 women (Intervention Group) ~36 women (Control Group)	BF education + nurse–midwife online counselling	Standard care	Exclusive BF, BF performance	↑ Exclusive BF and BF competence
Rodríguez-Gallego et al., 2024 [41]	Spain	RCT	~382 women	Postpartum midwife-led BF support groups	Usual postpartum care	Exclusive BF, self-efficacy	↑ Exclusive BF; sustained improvement in self-efficacy

**Table 3:** Key micronutrients in pregnancy: physiological roles, deficiencies, and outcomes.

Micronutrient	Primary Physiological Role	Common Deficiency	Maternal Outcomes	Neonatal Outcomes
Folate	DNA synthesis, cell division, neural tube closure	Inadequate periconceptional intake	Increased risk of anemia and pregnancy complications	Neural tube defects
Iron	Oxygen transport, erythropoiesis	Iron-deficiency anemia	Fatigue, anemia, obstetric complications	Low birth weight, impaired cognitive development
Iodine	Thyroid hormone synthesis, neurodevelopment	Borderline or low iodine intake	Thyroid dysfunction	Impaired neurodevelopment
Omega-3 (DHA)	Brain and retinal development	Low dietary intake	Increased risk of preterm birth	Suboptimal neurocognitive and visual development

## Data Availability

No new data were created or analyzed in this study. Data sharing is not applicable to this article.
